# Study of Potential Synergistic Effect of Probiotic Formulas on Acrylamide Reduction

**DOI:** 10.3390/ijms24054693

**Published:** 2023-02-28

**Authors:** Siu Mei Choi, Hongyu Lin, Weiying Xie, Ivan K. Chu

**Affiliations:** 1 Faculty of Science and Technology, Technological and Higher Education Institute of Hong Kong (THEi), Hong Kong; 2 Faculty of Science, The University of Hong Kong, Hong Kong

**Keywords:** synergistic effect, acrylamide, probiotic formula, toxin reduction

## Abstract

Acrylamide (AA) is a food processing contaminant commonly found in fried and baked food products. In this study, the potential synergistic effect of probiotic formulas in reducing AA was studied. Five selected probiotic strains (*Lactiplantibacillus plantarum* subsp. *plantarum* ATCC14917 (*L. Pl.*), *Lactobacillus delbrueckii* subsp. *bulgaricus* ATCC11842 (*L. B*.), *Lacticaseibacillus paracasei* subsp. *paracasei* ATCC25302 (*L. Pa*), *Streptococcus thermophilus* ATCC19258, and *Bifidobacterium longum* subsp. *longum* ATCC15707) were selected for investigating their AA reducing capacity. It was found that *L. Pl.* (10^8^ CFU/mL) showed the highest AA reduction percentage (43–51%) when exposed to different concentrations of AA standard chemical solutions (350, 750, and 1250 ng/mL). The potential synergistic effect of probiotic formulas was also examined. The result demonstrated a synergistic AA reduction effect by the probiotic formula: *L. Pl. + L. B.*, which also showed the highest AA reduction ability among the tested formulas. A further study was conducted by incubating selected probiotic formulas with potato chips and biscuit samples followed by an in vitro digestion model. The findings demonstrated a similar trend in AA reduction ability as those found in the chemical solution. This study firstly indicated the synergistic effect of probiotic formulas on AA reduction and its effect was also highly strain-dependent.

## 1. Introduction

Acrylamide (AA) is a low-molecular-weight organic compound with high polarity and medium activity. It has been considered a neurotoxin and potential carcinogen (Class II) harmful to the human body [1]. AA is formed in food materials by the Maillard reaction when amino acid reacts with reducing sugar at high temperatures, such as in deep fried and baked products [2]. In 2002, Swedish researchers found that plant foods rich in carbohydrates and low protein are prone to produce AA under high-temperature (>120 °C) cooking processes such as frying and baking [3,4]. Under certain conditions, AA will also be produced during the Maillard reaction of non-enzymatic browning of carbonyl compounds (reducing sugars) and amino compounds (proteins, amino acids) [5,6]. The World Health Organization (WHO) and the United Nations Food and Agriculture Organization (FAO) Joint Expert Committee on Food Additives (JECFA) conducted a systematic assessment of the hazards of AA in food, warning of potential hazards to human health. The intake control is 0.001 mg/kg per day [7].

The formation of acrylamide in food is a complex multi-stage reaction process, and its formation mechanism is not completely clear. At present, it was proposed that the formation of acrylamide in food is related to the Maillard reaction of asparagine and reducing sugar under high-temperature conditions. Therefore, in addition to processing conditions, the existence and content of reducing sugars and asparagine in food materials are important factors affecting the formation of acrylamide [2,8,9]. Many studies found that controlling the processing technology, such as soaking raw materials in warm water or citric acid solution, can reduce the AA content in fried French fries [10]. Lowering the processing temperature and reducing the heating time can also reduce the amount of AA produced [11]. During processing, adding inhibitors, such as asparaginase and sodium chloride, can significantly inhibit the AA content in food [11,12,13,14].

A previous study showed that an effective way to reduce acrylamide formation in bread is by using selected lactic acid bacteria (LAB) strains for fermentation of dough [15]. The purpose of this experiment is to prove the potential synergistic effect of probiotic formulas on reducing AA content.

In this research, representatives from three bacterial genera were evaluated, namely *Lactobacillus*, *Bifidobacterium,* and *Streptococcus*. They are the gut microbiota which can produce lactic acid and competitively inhibit the pathogenic organism [16]. The genus *Lactobacillus* is the largest genus of the LAB group traditionally associated with dairy products [17]. They can ferment carbohydrates, and together with *Streptococcus thermophilus*, they both act as major starter cultures in the dairy industry, while the addition of *Bifidobacterium longum* can improve the functional properties of fermented dairy products [18].

Besides the properties of promoting good digestion, boosting the immune system, maintaining a proper intestinal pH value, and competing with pathogens, there are several studies indicating the protective roles of LAB in mitigating the carcinogenic substances such as AA and aflatoxin B1 [19,20,21,22,23].

According to a recent review [23], mechanisms for reduction of AA can be proposed into two ways, one is the effect of asparaginase, and the other is substrate binding by probiotic bacteria. For the first mechanism, the AA formation can be inhibited by adding asparaginase in the raw material, reducing the content of asparagine and inhibiting the Maillard reaction. Thus, the amount of AA formed in the final product could be significantly reduced. This mechanism is applied to reducing AA formation during food processing, while the second mechanism can be applied in the post-processing stage to reduce AA after its formation.

For the second mechanism, the cell wall component peptidoglycan plays an important role in AA reduction. The peptidoglycan can bind to the AA due to its high affinity to alanine. They bound AA via hydrogen bonding, holding AA on the surface of the LABs and stopping its further biotransformation in the body [24]. Besides the peptidoglycan, some research also reported that other components of the cell walls such as teichoic acid also help in AA mitigation [25]. Based on the cell wall-binding mechanism, the dead LABs also have potential in AA reduction. Due to the wide exposure route of AA, more studies are needed for a further investigation of the mechanism of AA reduction and to excavate its potential for AA detoxification in daily life.

This study aims to examine the potential synergistic effect of AA reduction of LAB formulas which are commonly applied in dairy processing as starter cultures. The study first examined the AA reduction ability by a single strain of probiotic bacteria at different concentrations of AA chemical standard solution. Based on the results, five probiotic formulas were formed and their AA reduction ability was investigated. The probiotic formulas with the highest AA reduction ability were selected for further investigation in food samples and in an in vitro digestion model.

## 2. Results and Discussion

### 2.1. Acrylamide Reduction by Probiotics in Chemical Solution

#### 2.1.1. Acrylamide Reduction by Single Strains of Probiotic Bacteria

**Figure 1:**
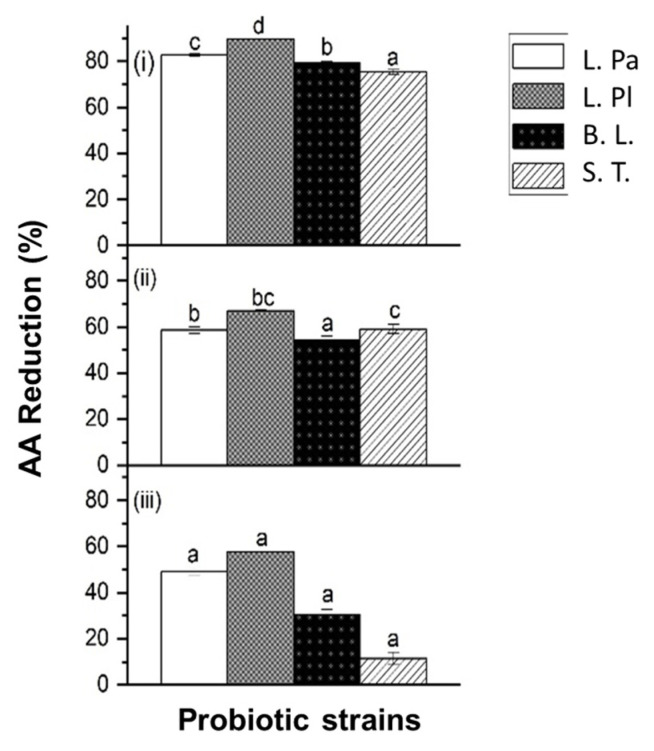
The acrylamide (AA) reduction ability of four single strains of probiotic bacteria: *Lacticaseibacillus paracasei* ATCC25302 (*L. Pa.*), *Lactiplantibacillus plantarum* ATCC14917 (*L. Pl.*), *Bifidobacterium longum* ATCC15707 (*B. L.*), and *Streptococcus thermophilus* ATCC19258 (*S. T.*), when exposed to (**i**) 350 ng/mL, (**ii**) 750 ng/mL, and (**iii**) 1250 ng/mL AA solutions. Probiotic concentration was 10^9^ CFU/mL. Incubated at 37 °C for 4 h at pH 6.5–7.0. Expressed as AA reduction percentage. Different characters in the same panel indicate significant differences, whereas the same character indicates not significant.

As shown in Figure 1, *L. Pa., L. Pl., B. L.,* and *S. T.* at a cell population of 10^9^ CFU/mL demonstrated significant reduction abilities on AA when exposed to different concentrations of AA chemical solutions (*p* < 0.05). At the same cell concentration, the four strains showed different AA reduction abilities, ranging from 75% to 89% when exposed to the same concentration of AA (350 ng/mL). The data showed that *L. Pl.* demonstrated the highest AA reduction percentage among the tested strains. It was observed that *L. Pl.* showed the highest reduction percentages of 89% and 67% when exposed to AA solutions of 350 ng/mL and 750 ng/mL, respectively. These findings indicated that different bacterial strains have different AA reduction abilities. Hence, the AA reduction ability of probiotics is strain-specific. Similar results were also observed in a previous study [21].

In addition, the effect of different AA concentrations on the AA reduction ability of probiotics was also obtained. All tested probiotic strains showed an ability to reduce AA and the reduction percentage varied when exposed at different AA concentrations (Figure 1). The highest AA reduction ability was obtained when exposed to the low AA concentration of 350 ng/mL. As shown in Figure 1, *L. Pa., L. Pl., B. L.,* and *S. T.* (10^9^ CFU/mL) showed a significant reduction (*p* ≤ 0.05) in their abilities to reduce AA when the AA concentration was increased from 350 ng/mL to 750 ng/mL. Moreover, the AA reduction abilities of tested strains had significantly (*p* ≤ 0.05) declined when the AA concentration was further increased up to 1250 ng/mL.

This result might be explained by the potential reduction mechanism of the probiotic, namely, the physical binding mechanism of the bacterial cell wall [23]. Peptidoglycan is the major cell wall mass of LAB. The structure of glycan chains of repeating *N*-acetylglusamine and *N*-acetylmuramic acid residues cross-link via the peptide side chain [26]. This physical binding mechanism was also applied in other toxic substances, such as polyaromatic hydrocarbon and mycotoxins, to explain the toxin-removing ability of LAB [27,28]. The physical binding effect was affected by several factors, including incubation time and toxin concentration. When the acrylamide concentration increased, the peptidoglycan cell wall tended to become saturated with AA and showed a decrease in the ability to remove AA [20]. The current study also demonstrated similar observations that can be explained by the physical binding mechanism.

**Figure 2:**
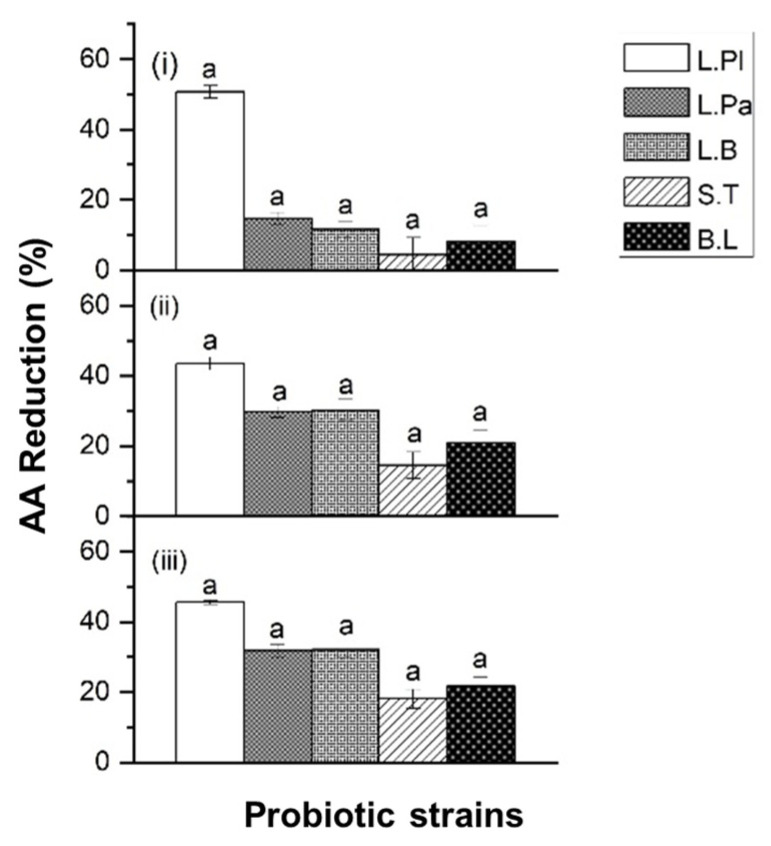
The acrylamide (AA) reduction ability of five strains of probiotic bacteria: *Lacticaseibacillus paracasei* ATCC25302 (*L. Pa.*), *Lactiplantibacillus plantarum* ATCC14917 (*L. Pl.*), *Lactobacillus bulgaricus* ATCC11842 (*L. B.*), *Bifidobacterium longum* ATCC15707 (*B. L.*), and *Streptococcus thermophilus* ATCC19258 (*S. T.*), when exposed to (**i**) 350 ng/mL, (**ii**) 750 ng/mL, and (**iii**) 1250 ng/mL AA solutions. Probiotic concentration was 10^8^ CFU/mL. Incubated at 37 °C for 4 h at pH 6.5–7.0. Expressed as AA reduction percentage. Different characters in the same panel indicate significant differences, whereas the same character indicates not significant.

When the bacterial cell concentration was adjusted to 10^8^ CFU/mL (Figure 2), *L. Pl.* demonstrated the highest AA reduction percentages (43–51%) among the 5 tested probiotic strains when exposed to different AA concentrations of 350 ng/mL, 750 ng/mL, and 1250 ng/mL, respectively. As shown in Figure 2, it was seen that upon increasing the AA concentration, *L. Pl.* significantly (*p* ≤ 0.05) decreased its AA reduction abilities. In general, all selected probiotic strains exhibited AA reduction abilities at different AA concentrations. Different probiotic strains showed a different AA reduction performance. This may be explained by different probiotic strains functioning differently with variations in the contents of carbohydrates and certain amino acids of their bacterial cell wall [19,21].

As shown in Figure 1 and Figure 2, the selected *Lactobacillus* genus exhibited a more efficient AA reduction ability than two other genera under the testing conditions. *L. Pl.* yielded the best results, while *L. B.* and *L. Pa.* showed similar AA reduction abilities. Some previous studies indicated that the AA absorption effect was related to the roughness of the cell wall and the surface hydrophobicity [22], as well as the bonding interaction with the teichoic acid content of the cell wall [19]. Hence, the difference in the reduction ability of test strains is due to the difference in their cell wall composition.

In addition, the percentage of AA reduction was increased when the probiotic cell concentration increased from 10^8^ CFU/mL to 10^9^ CFU/mL. With the 350 ng/mL AA solution, *L. Pl.* caused a dramatic increase in the AA reduction percentage from 51% to 89% when the cell concentration was increased from 10^8^ CFU/mL to 10^9^ CFU/mL. Similarly, with the 750 ng/mL AA solution, *L. Pl.* increased its AA reduction percentage from 43% to 67% when the cell concentration was increased from 10^8^ CFU/mL to 10^9^ CFU/mL (Figure 1 and Figure 2).

Hence, the current results also demonstrated that the effect of AA reduction by probiotic bacteria is strain-, cell concentration-, and AA concentration-dependent.

#### 2.1.2. Acrylamide (AA) Reduction by Probiotic Formulas in Chemical Solution

**Figure 3:**
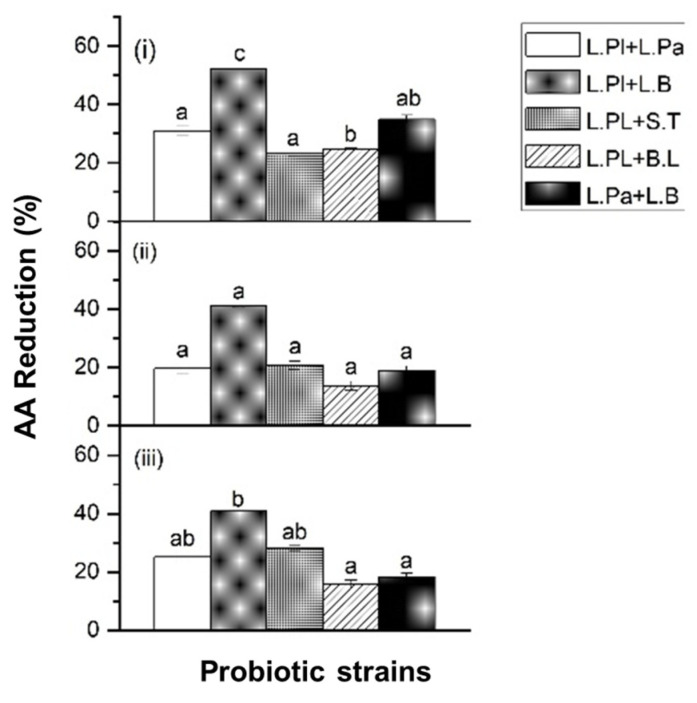
The acrylamide (AA) reduction ability of five formulas of probiotic bacteria: *Lactiplantibacillus plantarum* ATCC14917 (50%) + *Lacticaseibacillus paracasei* ATCC25302 (50%) (*L. Pl.* + *L. Pa.*), *Lactiplantibacillus plantarum* ATCC14917 (50%) + *Lactobacillus bulgaricus* ATCC11842 (50%) (*L. Pl.* + *L. B.*), *Lactiplantibacillus plantarum* ATCC14917 (50%) + *Streptococcus thermophilus* ATCC19258 (50%) (*L. Pl.* + *S. T.*), *Lactiplantibacillus plantarum* ATCC14917 (50%) + *Bifidobacterium longum* ATCC15707 (50%) (*L. Pl.* + *B. L.*), and *Lacticaseibacillus paracasei* ATCC25302 (50%) + *Lactobacillus bulgaricus* ATCC11842 (50%) (*L. Pa.* + *L. B.*), when exposed to (**i**) 350 ng/mL, (**ii**) 750 ng/mL, and (**iii**) 1250 ng/mL AA solutions. Probiotic concentration was 10^8^ CFU/mL. Incubated at 37 °C for 4 h at pH 6.5–7.0. Expressed as AA reduction percentage. Different characters in the same panel indicate significant differences, whereas the same character indicates not significant.

Based on the results of the AA reduction, five probiotic formulas (10^8^ CFU/mL) were formed to evaluate the potential synergetic reduction effect on AA. Due to the highest AA reduction ability, *L. Pl.* was selected and combined with the other strains. As shown in Figure 3, the results showed that different probiotic formulas had a significant effect on AA reduction when exposed to different AA solutions. The probiotic formula of *L. Pl. + L. B.* showed the highest AA reduction percentage among the selected formulas (Figure 3). The result also demonstrated a synergistic reduction effect on AA by this probiotic formula (*L. Pl. + L. B.*). Before combining with *L. Pl.*, *L. B.* alone showed its AA reduction rate of 12%, 30%, and 32% at 350 ng/mL, 750 ng/mL, and 1250 ng/mL, respectively. After combining with *L. Pl.*, the AA reduction rate increased to 52%, 41%, and 41% at 350 ng/mL, 750 ng/mL, and 1250 ng/mL, respectively. *L. Pl.* can hydrolyze asparagine, the precursor of acrylamide, to generate aspartic acid and ammonia, thereby inhibiting the formation of acrylamide to a certain extent [21].

The combination of probiotic strains also improved the efficacy on AA reduction. In the single-strain condition, *S. T.* showed a weaker reduction ability than *B. L.* However, a higher reduction ability was observed when *S. T.* was combined with *L. Pl.* when compared with *B. L.* combined with *L. Pl.*, as shown in Figure 2 and Figure 3. The co-culture concept of different strains led to a surprising discovery, such as the new metabolites or the metabolism pathway modulation [29]. The combination of different probiotic single strains also showed its potential synergistic effect on acrylamide detoxification. However, this synergistic effect was also strain-dependent.

The synergistic effect of combined probiotics was also reported in another study [30], which indicated the significant effects of combined probiotics to prevent colon cancer. According to previous findings [31], probiotics and their cell wall extracts could have anticancer ability, and the synergistic effect was found when probiotics were combined with other bio-functional compounds from cranberry juice. Another study [32] also found that the combination of prebiotics and probiotics had a synergistic effect because it promoted the growth of existing beneficial bacteria in the colon, and synbiotics also played a role in improving the survival, implantation, and growth of newly added probiotic strains in rats. One study showed that the probiotic efficiency of probiotic bacteria may be different if they were used in different hosts [33].

### 2.2. Acrylamide (AA) Reduction by Probiotic Formulas in Food Matrices

**Figure 4:**
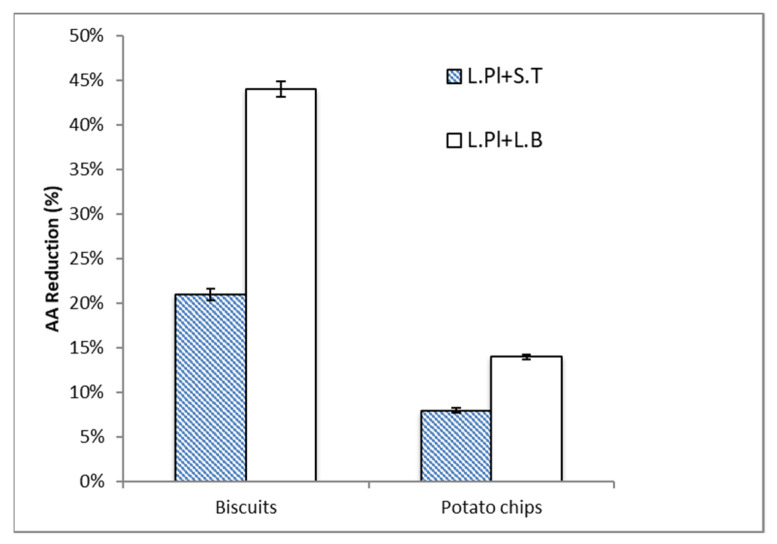
The acrylamide (AA) reduction ability of two formulas of probiotic bacteria: *Lactiplantibacillus plantarum* ATCC14917 (50%) + *Streptococcus thermophilus* ATCC19258 (50%) (*L. Pl.* + *S. T.*) and *Lactiplantibacillus plantarum* ATCC14917 (50%) + *Lactobacillus bulgaricus* ATCC11842 (50%) (*L. Pl.* + *L. B.*), when exposed to biscuits and potato chips. Probiotic concentration was 10^8^ CFU/mL. Incubated at 37 °C for 4 h at pH 6.5–7.0. Expressed as AA reduction percentage. Data are the mean of three replicates.

In this study, the AA content of the selected food samples, biscuits and potato chips, were detected as 55.1 ng/g and 217.0 ng/g, respectively. In Figure 4, the AA reduction ability of two probiotic formulas was investigated under two different food matrices (biscuits and potato chips). The AA content of the selected food samples was significantly reduced. The result showed that the percentage of AA reduction in biscuits was higher than that in potato chips by both probiotic formulas (*L. Pl. + S. T.* and *L. Pl. + L. B.*) at 10^8^ CFU/mL. Similar to the results in chemical solution, the formula *L. Pl. + L. B.* showed the higher AA reduction ability. The AA reduction percentage by the formula of *L. Pl. + L. B.* was two times higher than that by *L. Pl. + S. T.* in both biscuit and potato chip samples.

Different food compositions in food matrices may affect the effect of probiotics. Previous findings [22] suggested that increasing the roughness of cell walls and increasing the surface hydrophobicity of cells enhanced the adsorption ability of AA. Furthermore, the C-O, C=O, and N-H groups, which were related to the protein and peptidoglycan contents of the cell wall, were evidently involved in AA adsorption.

Therefore, different AA reduction percentages by probiotic formulas were obtained in different food models.

### 2.3. Acrylamide (AA) Reduction by Probiotic Formulas in Food Matrices under In Vitro Digestion

**Figure 5:**
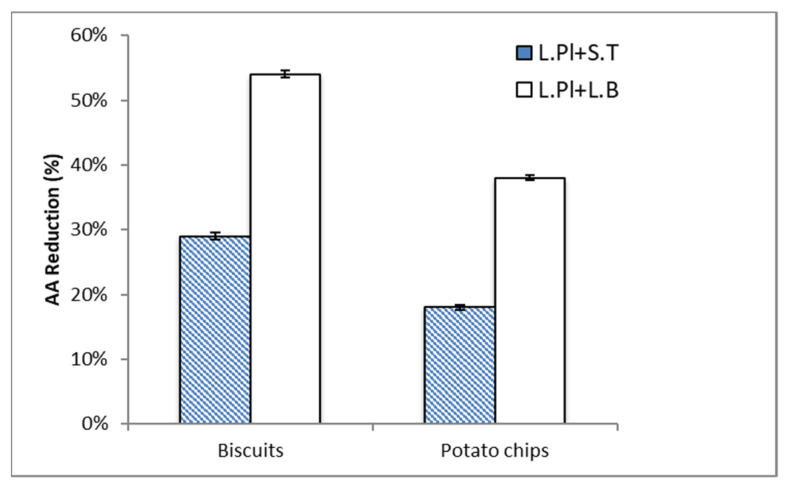
The acrylamide (AA) reduction ability of two formulas of probiotic bacteria: *Lactiplantibacillus plantarum* ATCC14917 (50%) + *Streptococcus thermophilus* ATCC19258 (50%) (*L. Pl.* + *S. T.*) and *Lactiplantibacillus plantarum* ATCC14917 (50%) + *Lactobacillus bulgaricus* ATCC11842 (50%) (*L. Pl.* + *L. B.*), when exposed to biscuits and potato chips under in vitro digestion. Probiotic concentration was 10^8^ CFU/mL. Data are the mean of three replicates.

In Figure 5, the AA reduction ability of two probiotic formulas was further investigated in two different food matrices (biscuits and potato chips) under a simulated digestion model. Similar trends were obtained in food matrices with or without digestive conditions. The result showed that both probiotic formulas: *L. Pl. + S. T.* and *L. Pl. + L. B.* (10^8^ CFU/mL), caused a higher AA reduction percentage in the biscuit food model under the digestive condition when compared with that in potato chips. To compare the efficacy of the probiotic formulas, *L. Pl. + L. B.* showed a higher AA reduction ability than *L. Pl. + S. T.* Based on the data, the AA reduction ability of probiotic formulas in food matrices was significantly increased under the in vitro digestion condition. In the potato chips model, the AA reduction percentage by *L. Pl. + L. B.* was increased from 14% to 38% under the in vitro digestion condition (Figure 4 and Figure 5).

The use of in vitro simulated digestion model could be considered as a useful tool to estimate the bioavailability of acrylamide under testing conditions. It considered not only the influence of food intrinsic factors (structure, composition, nutrients’ interactions, etc.) but also extrinsic factors associated with the physiological process (gastric and intestinal pH, transit time, enzymatic activities, etc.) [25]. The current study may indicate the ability of probiotic formulas to reduce the bio-accessibility of food toxicants under digestive conditions. Previous studies also reported a similar toxin removal capacity of probiotic bacteria under a simulated digestion condition [34,35,36].

## 3. Materials and Methods

### 3.1. Probiotic Strains and Culture Preparation

*Lactobacillus delbrueckii* subsp. *bulgaricus* (ATCC^®^ 11842™) (*L. B.*), *Lacticaseibacillus paracasei* subsp. *paracasei* (ATCC^®^25302™) (*L. Pa.*), *Lactiplantibacillus plantarum* subsp. *plantarum* (ATCC^®^14917™) (*L. Pl.*), *Streptococcus thermophilus* (ATCC^®^19258™) (*S. T.*), and *Bifidobacterium longum* subsp. *longum* (ATCC^®^15707™) (*B. L.*) were used. *S. T.* was activated in Brain Heart Infusion (BHI) (MEKESSON, Irving, USA) by aerobic cultivation, while the other four strains were activated by de Man Rogosa Sharpe (MRS) broth (Thermo Fisher Scientific Inc., Waltham, MA, USA) under anerobic cultivation. All the tubes were put into the anaerobic jar with an anaerobic atmosphere generation bag. The bacteria were activated in the incubator at 37 °C for at least 24 h to reach maximum growth. Subsequently, subcultures were performed prior to the experiment. For each subculture, an aliquot from the last subculture was added to 100 mL of sterile MRS broth and incubated at 37 °C for at least 24 h to achieve maximum growth. Optical density (OD600) of bacterial strains was measured by a UV-vis single-beam spectrophotometer to obtain the growth curves, and the pour plating method was used to determine the cell concentration (CFU value). All agar plates were incubated under 37 °C for 48 h. The number of colonies was counted for CFU determination.

The bacteria were collected by centrifugation at 2100× *g* for 10 min and washed twice with sterile phosphate buffer saline (PBS) (Sigma-Aldrich, St. Louis, MO, USA). The pellets were re-suspended in sterile PBS to obtain the primary working cultures (10^9^ CFU/mL). All prepared working cultures were temporarily stored at 4 °C prior to analysis [37].

### 3.2. Reagents

Methanol, hydrochloric acid (HCl, 1 M), sodium hydroxide (NaOH, 1 M), sodium chloride (NaCl), potassium chloride (KCl), sodium bicarbonate (NaHCO_3_), sodium dihydrogen phosphate (NaH_2_PO_4_), sodium sulfate (Na_2_SO_4_), potassium thiocyanate (KSCN), calcium chloride dihydrate (CaCl_2_∙2H_2_O), ammonium chloride (NH_4_Cl), potassium dihydrogen phosphate (KH_2_PO_4_), magnesium chloride (MgCl_2_), urea, uric acid, mucin, bovine serum albumin (BSA), pepsin, pancreatin, lipase, α-amylase, and bile were used. All reagents were of analytical grade and all organic solvents were of LC/MS HPLC grade, unless otherwise stated. Acrylamide standard (>99.5%) and ^13^C_3_-acrylamide as internal standards (500 mg/L in acetonitrile) were purchased from Chem Service Inc. (West Chester, PA, USA) and Sigma-Aldrich (St. Louis, MO, USA), respectively. Oasis HLB cartridge (200 mg, 6 cc) was purchased from Waters Corporation (Milford, MA, USA) and Bond Elut Accuat cartridge (200 mg, 3 cc) was purchased from Agilent Technologies, Inc. (Santa Clara, CA, USA) [37].

The AA standard stock solution (1000 μg/mL) and ^13^C_3_-AA internal standard stock solution were prepared. A five-point calibration curve was constructed using the AA working solutions (30 to 500 ng/mL). Various concentrations of AA chemical solutions (350, 750, 1250 ng/mL) were prepared for the experiments on AA reduction ability. All standard solutions were prepared and stored at 4 °C.

### 3.3. Acrylamide Reduction Ability of Single Probiotic Strains and Probiotic Formulas in Standard Chemical Solutions

Selected single probiotic strains (*L. Pl., L. Pa.., L. B., S. T., B. L.*) were incubated with various concentrations of AA chemical solutions under 10^9^ and 10^8^ CFU/mL cell concentrations. The mixtures were briefly vortexed and then incubated at 37 °C for 4 h (close to the total incubation time of the in vitro digestion model) with gentle rotation (55 rpm). After incubation, the mixtures were centrifuged at 20,000× *g* for 10 min at 25 °C. The content of AA in the supernatant was determined by LC-MS analysis, as described in Section 3.6. The AA reduction percentage of single probiotic strains was calculated.

According to the performance of the single probiotic strains on AA reduction, the probiotic formulas (10^8^ CFU/mL) were formed as below and the same procedures were applied to obtain AA reduction percentages of different probiotic formulas in chemical solutions.

(1) *Lactiplantibacillus plantarum* ATCC14917 (50%) + *Lacticaseibacillus paracasei* ATCC25302 (50%) (*L. Pl.* + *L. Pa.*).
(2) *Lactiplantibacillus plantarum* ATCC14917 (50%) + *Lactobacillus bulgaricus* ATCC11842 (50%) (*L. Pl.* + *L. B.*).
(3) *Lactiplantibacillus plantarum* ATCC14917 (50%) + *Streptococcus thermophilus* ATCC19258 (50%) (*L. Pl.* + *S. T.*).
(4) *Lactiplantibacillus plantarum* ATCC14917 (50%) + *Bifidobacterium longum* ATCC15707 (50%) (*L. Pl.* + *B. L.*).
(5) *Lacticaseibacillus paracasei* ATCC25302 (50%) + *Lactobacillus bulgaricus* ATCC11842 (50%) (*L. Pa.* + *L. B.*).

### 3.4. Acrylamide Reduction Ability of Probiotic Formulas in Selected Food Matrices

Potato chips and soda biscuits were selected as food samples. These two selected food samples were found to contain a relatively high AA content as AA were the common processing-induced contaminants formed during baking and frying production processes.

According to the results of the AA reduction ability of probiotic formulas in part 1 (Section 3.3), two probiotic formulas with the potential synergetic effect were selected for further incubation in food samples. Spiked homogeneous food sample (1.0 g) and 0.5 mL of working cultures of probiotic formulas or PBS solution (control) were added to 4.5 mL of sterile PBS solution, incubating at 37 °C for 4 h with gentle rotation at 55 rpm. The resultant probiotic concentration remained at 10^8^ CFU/mL. After incubation, the mixtures were centrifuged at 20,000× *g* for 10 min at 25 °C. The incubated samples were then centrifuged, and the supernatant was cleaned up by solid-phase extraction (SPE) and subjected to LC-MS analysis to determine the AA content.

### 3.5. Acrylamide Reduction Ability of Probiotic Formulas under In Vitro Digestion Model

To determine the efficacy of probiotic formulas to reduce AA content in food matrices under a stimulated digestion condition, the procedures of Choi et al. [37] were followed. The samples for in vitro digestion were prepared as below. Here, 1 g of grounded food sample was added to 5 mL of PBS (control) or working cultures of selected probiotic formulas: (1) *Lactiplantibacillus plantarum* ATCC14917 coupled with *Lactobacillus bulgaricus* ATCC11842 and (2) *Lactiplantibacillus plantarum* ATCC14917 coupled with *Streptococcus thermophilus* ATCC19258 (10^8^ CFU/mL).

Digestive fluids (Table S1—Supplementary Materials) were added according to the volume ratio of 1 chemical solution/food:1.3 saliva:2.6 gastric juice:2.6 duodenum juice:1.3 bile:0.44 NaHCO_3_. All digestive fluids were adjusted to 37 ° C before use. Specifically, 1.3 µL of saliva was added to the mixture and the mixtures were incubated at 37 °C/55 rpm for 5 min in a shaking incubator. This was followed by the adding 2.6 mL of gastric juice and adjusting the pH of the mixtures to 2.5–3.0 with 1 M HCl or NaOH. The mixtures were then incubated at 37 °C and 55 rpm for 2 h. Next, 2.6 mL of duodenum juice, 1.3 mL of bile, and 0.44 mL of NaHCO_3_ (1.0 M) were added simultaneously, and the pH was adjusted to 6.5–7.0 with 1.0 M HCl or NaOH. Then, the mixtures were incubated at 37 °C and 55 rpm for 2 h. At the end of the digestion process (stomach or small intestine compartment), two aliquots from the upper part of the mixture were centrifuged at 20,000× *g* for 10 min at 25 °C. Finally, the resulted supernatants were cleaned-up by SPE using the same procedures in Section 3.4 and subjected to LC-MS analysis of AA.

### 3.6. Solid-Phase Extraction

To extract AA from the food matrix, solid-phase extraction was used. The Oasis HLB cartridge (Water, Milford, MA, USA) was pre-conditioned with 3.5 mL of methanol followed by 3.5 mL of Milli-Q water, while Bond Elut Accuat cartridge (Agilent Technologies, Santa Clara, CA, USA) was pre-conditioned with 2.5 mL of methanol and 2.5 mL of Milli-Q water, sequentially. After that, 1.5 mL of the clear supernatant was loaded onto the HLB cartridge, followed by washing with 0.8 mL of Milli-Q water (all discarded). Then, the AA was eluted with 3 mL of 50% methanol from the HLB cartridge to the Bond Elut Accuat cartridge. Finally, 25 μL of ^13^C_3_-AA (40 ng/mL, internal standard) was added to the 3 mL of effluents and then filtered through a 0.45 μm filter into a LC-MS vial for LC-MS analysis. The recovery rate of the two food samples was evaluated as:

(1) $$\mathrm{Recovery}\;\mathrm{rate}\;(\%)=\frac{Conc.\;of\;AA\;in\;spiked\;sample-Conc.\;of\;AA\;in\;non\text{-}spiked\;sample}{Conc.\;of\;spiked\;AA}$$

### 3.7. LC-MS Method for the Analysis of Acrylamide

The AA analysis was performed with an Agilent 1260 Infinity II LC-MS system coupled to an Agilent 6120 Single-Quad MS system (Agilent Technologies Inc., Santa Clara, CA, USA) with an electrospray-type ionization source. The column of the LC-MS system used was a Resiek Ultra AQ C18 column (3 µm, 100 mm × 2.1 mm) (Bellefonte, PA, USA).

The sample was separated by the mobile phase (aqueous 0.2% acetic acid and 1% methanol) for 7 min at 0.200 mL/min with a 10 µL injection volume after 1 min post-time to equilibrate the column. The column oven temperature was set at 35 °C and the electrospray was operated in positive ion mode. The conditions used in the ionization source were: 250 °C at 12.0 L/min for the drying gas (N_2_), a nebulizer pressure of 35 psig, and a capillary voltage of 3000 V. AA was determined using the Selective Ion Monitoring mode (SIM), monitoring the ions *m*/*z* 72.0 for AA and 75.0 for ^13^C_3_-AA (internal standard).

By using a five-point calibration curve, the concentrations of AA in the control and samples were determined and then the percentage of AA reduction was obtained using the following equation:

(2) $$\mathrm{AA}\;\mathrm{reduction}\;(\%)=\frac{Conc.\;of\;AA\;in\;control\;(PBS)-Conc.\;of\;AA\;in\;sample}{Conc.\;of\;AA\;in\;control\;(PBS)}$$

### 3.8. Statistical Analysis

Results are expressed as mean ± SEM. Statistical analyses were performed using SPSS Statistics 22. Results were considered statistically significant when *p* < 0.05 for specific *p*-values.

## 4. Conclusions

This study demonstrated the AA removal ability of probiotic bacteria, whereby the reduction ability depends on the strains of single probiotic bacteria and probiotic formulas. The current findings showed a potential synergistic effect in some probiotic formulas which could enhance the efficacy of AA reduction. Among the tested probiotic combinations, the best probiotic formula was *Lactiplantibacillus plantarum* ATCC14917 (50%) *+ Lactobacillus bulgaricus* ATCC11842 (50%) (*L. Pl. + L. B.*)*,* which can cause a 41–52% AA reduction percentage in different AA chemical solutions. This formula also demonstrated its ability to reduce AA in both food samples of biscuits and potato chips with or without the in vitro digestion condition. Our findings suggested that specific probiotic formulas could be applied to reduce the dietary acrylamide in the gastrointestinal tract and thereby decrease its potential risk of toxic effects in the human body.

## Data Availability

Not applicable.

## Supplementary Materials

The following supporting information can be downloaded at: https://www.mdpi.com/article/10.3390/ijms24054693/s1.
