# Supplementary material for: Study of Potential Synergistic Effect of Probiotic Formulas on Acrylamide Reduction
Source: Int J Mol Sci. 2023 Feb 28;24(5):4693. doi: 10.3390/ijms24054693 (PMC10003183; doi:10.3390/ijms24054693)
Supplement: Supplementary file 1 [file ijms-24-04693-s001.zip › ijms-2167038-supplementary.pdf]

Table S1. Constituents of the various synthetic digestion fluids of the *in vitro* digestion model (per liter)

Digestive juice	Saliva	Gastric Juice	Duodenal Juice	Bile Juice
Inorganic	0.3 g NaCl<br>0.9 g KCl<br>1.7 g $NaHCO_3$<br>0.9 g NaH_2PO_4<br>0.57 g $NaSO_4$<br>0.2 g KSCN	2.75 g NaCl<br>0.82 g KCl<br>0.27 g NaH_2PO_4<br>0.4 g $CaCl_2.H_2O$<br>0.31 g NH_4Cl<br>6.5 mL HCl (37%)	7.01 g NaCl<br>0.56 g KCl<br>3.39 g $NaHCO_3$<br>0.08 g KH_2PO_4<br>0.05 g $MgCl_2$<br>0.18 mL HCl (37%)	5.26 g NaCl<br>0.38 g KCl<br>5.79 g $NaHCO_3$<br>0.15 mL HCl (37%)
Organic	0.2 g urea	0.65 g glucose<br>0.02g glucuronic acid<br>0.33 g glucosamine hydrochloride<br>0.085 g urea	0.1 g urea	0.25 g urea
Add to the mixture organic+inorganic solution	290 mg a-amylase<br>15 mg uric acid<br>25 mg mucin	1 g BSA<br>2.5 g pepsine<br>3 g mucin	0.2 g $CaCl_2 \cdot 2H_2O$<br>1 g BSA<br>9 g pancreatin<br>1.5 g lipase	0.22 g $CaCl_2 \cdot 2H_2O$<br>1.8 g BSA<br>30 g Bile
pH	7.9±0.2	1.30±0.02	8.8±0.2	8.2±0.2

Abbreviations

HCl	hydrochloric acide
NaOH	sodium hydroxide
NaCl	sodium chloride
KCl	potassium chloride
$NaHCO_3$	sodium bicarbonate
NaH_2PO_4	sodium dihydrogen phosphate
Na_2SO_4	sodium sulfate
KSCN	potassium thiocyanate
$CaCl_2 \cdot 2H_2O$	calcium chloride dihydrate
NH_4Cl	ammonium chloride
KH_2PO_4	potassium dihydrogen phosphate
$MgCl_2$	magnesium chloride
BSA	bovine serum albumin
